# Urine complement analysis implies complement activation is involved in membranous nephropathy

**DOI:** 10.3389/fmed.2025.1515928

**Published:** 2025-02-13

**Authors:** Yingxue Xu, Yi Li, Yong Zhang, Guisen Li

**Affiliations:** ^1^Department of Nephrology and Nephrology Institute, Sichuan Provincial People’s Hospital, School of Medicine, University of Electronic Science and Technology of China, Chengdu, China; ^2^Department of Nephrology, The Affiliated Hospital of Southwest Medical University, Luzhou, China; ^3^Department of Nephrology and Institutes for Systems Genetics, West China Hospital, Sichuan University, Chengdu, China

**Keywords:** membranous nephropathy, complement, proteomics, histopathology, clinical remission

## Abstract

**Background:**

The onset and progression of membranous nephropathy (MN) have been associated with complement activation, yet the overall characteristics of this activation in the kidneys remain unclear. In our study, we utilized urine proteomic data to investigate the features of complement activation. We examined the relationship between urine complement components and both clinicopathological features and clinical outcomes in patients with MN.

**Methods:**

Differential expression proteins (DEPs) analysis was performed using proteomic data from urine samples collected from 50 patients with MN, 50 patients with IgA nephropathies (IgAN), and 72 healthy controls (HC). Then, Gene Ontology and Kyoto Encyclopedia of Genes and Genomes analyses were carried out on the DEPs identified in MN. We further investigated the differentially expressed urinary complement proteins in MN patients, exploring their relationships with clinicopathological features and clinical remission. Next, 11 representative complements were selected for validation. Immunohistochemistry and immunofluorescence techniques were employed to compare the expression of CD59 and C5b-9 in renal tissues from MN patients, with analyses conducted on both the clinical remission group and the no remission group (*n* = 6 in each group).

**Results:**

Total 1,427 differentially expressed proteins were identified between the MN and HC groups. KEGG pathway analysis showed significant enrichment of these DEPs in the complement-activated pathway within the MN group. Additionally, a correlation was found between proteinuria and the levels of 27 urinary complement proteins. Notably, Collectin12 (collec12) and C1s were positively correlated with tubular atrophy/interstitial fibrosis (TIF) and monocyte infiltration. Furthermore, urine CD59 emerged as a predictor of clinical remission. Lower deposition of C5b-9 in renal tissue and higher expression of CD59 were detected in clinical remission group than non-remission group.

**Conclusion:**

In patients with MN, abnormal levels of complement components in urine are commonly observed. Currently, the use of complement inhibitors has brought new hope for the treatment of MN. The factor B inhibitor LNP023 and the factor D inhibitor BCX9930 are undergoing clinical trials for the treatment of MN. Our study indicates that complement abnormalities could serve as clinical biomarkers for tracking the progression of MN, predicting clinical remission, and guiding targeted complement therapy for those affected.

## Introduction

In recent years, membranous nephropathy (MN) has emerged as the leading cause of nephrotic syndrome in adult patients, with approximately 40% of cases progressing to end-stage renal disease within 5 to 15 years (1). In primary MN, endogenous glomerular antigens, such as the M-type phospholipase A2 receptor (PLA2R) and the 7A-containing platelet-responsive protein type 1 structural domain (THSD7A), bind to antibodies, forming immune complexes in the glomerulus. These immune complexes trigger the complement cascade, primarily activating the lectin-complement pathway, which is one of the three pathways of complement activation. It is activated by recognition of non-self or altered self glycan structures by mannose-binding lectin (MBL), collectin-10, collectin-11 or ficolins (ficolin-1, -2, -3). Activation of complement leads to the formation of the membrane attack complex (MAC, C5b-9), which is the end product of complement activation that inserts directly into the podocyte membrane (2). Lead to podocyte damage, disruption of the glomerular filtration barrier, and ultimately to proteinuria in patients with MN (3). Notably, components from all three complement pathways can be detected in renal biopsies of patients diagnosed with MN.

Although the presence of activated complements in the injured glomeruli of patients with MN is well-established, the overall characteristics, initiating pathways, and pathogenic roles of complement activation in this condition remain unclear. Various studies have demonstrated that complement activation in renal tissues of MN is closely associated with renal injury and poor outcomes. Glomerular C3 staining intensity has been found to correlate with proteinuria (4, 5) and extensive glomerular C3 deposition can serve as a predictor of renal failure in patients with MN (6). Additionally, C5b-9 deposition has been linked to lower renal survival in MN (7), as well as the presence of MBL and C4d deposits is associated with increased renal interstitial fibrosis and no remission in patients with MN (8–10). Recent studies utilizing laser microdissection and mass spectrometry (MS/MS) have detected abundant complement proteins such as C3, C4, C5, C6-9, complement factor H (CFH), vitronectin (VTN), and clusterin (CLU) in the renal tissues of MN patients (11, 12). However, it is not possible to repeatedly obtain and use renal tissue for tracking complement activation.

Previous studies have shown that activated complement can be found in both blood and urine, and its levels correlate with clinical and pathological features, as well as disease progression in patients with MN. The presence of circulating MBL-associated serine proteases-1/2 (MASP-1/2), MBL, C3, C4, C5a, CFH, and complement factor B (CFB) has been found to be linked with disease progression according to several studies (13–18). Urinary levels of MBL, C4d, and C5a have shown positive correlations with urinary protein (19). Additionally, Studies have demonstrated that high levels of urinary C3dg and C5b-9 are significantly associated with MN, and patients with high urinary C5b-9 have exhibited an unstable clinical course (20). It has also been noted that persistent C5b-9 excretion is linked to poor clinical outcomes (21, 22). Several clinical trials targeting the complement system are currently underway in patients with MN, following these findings.

The overall expression profile of complement components in the blood and urine of patients with MN has not been thoroughly explored in previous studies, which mainly focus on individual complement proteins. This study aims to analyze the complement component profile in the urine of MN patients using proteomic data obtained from liquid chromatography–tandem mass spectrometry (LC–MS/MS). Additionally, we will examine the relationship between urinary complement levels, clinicopathological features, and clinical remission in MN patients. The findings from this study could offer valuable insights for monitoring complement activation, evaluating complements in clinical prognosis, and developing complement-targeted therapies for MN patients.

## Materials and methods

### Participants and study design

A total of 248 participants who visited Sichuan Provincial People’s Hospital from January 1, 2018 to December 31, 2023 were included in this study. We divided 248 participants into a discovery cohort and a validation cohort. The discovery cohort consisted of 80 patients with MN, 50 patients with IgA nephropathy (IgAN), and 68 healthy controls (HC) to explore the overall characterization of urinary proteins by LC–MS/MS detection of urinary proteomic data. The validation cohort included 80 patients with MN and was used to validate the association of urinary complement proteins with clinicopathologic features and clinical remission. A total of 248 urine samples from subjects with MN (*n* = 130), IgAN (*n* = 50), and HC (*n* = 68) were collected in tubes according to standard operating procedures. Inclusion criteria: (1) MN was diagnosed by renal biopsy. (2) Clinical and pathological data were accessible. (3) Urine specimens were collected in the early morning of renal biopsy according to standard procedures. (4) Patients agreed to use urine specimens and renal pathological specimens remaining for experiments. Exclusion criteria: (1) Secondary MN such as secondary to autoimmune disease, diabetes mellitus, tumors, infections, medications, etc. (2) Treatment with hormones, immunosuppressants, or biologics, etc. prior to the renal biopsy. (3) Age less than 18 years old. (4) Pregnancy. (5) Patient did not agree to participate in the study. The study adhered to the principles of the Declaration of Helsinki and obtained approval from the Medical Ethics Committee of Sichuan Provincial People’s Hospital. The written informed consent was obtained from all participants.

### The standard procedure for urine collection and mass spectrometric analysis

The standard procedure for urine collection is as follows: a 50 mL centrifuge tube is used to collect the clean midstream urine from the patient in the morning for renal biopsy, in a volume of approximately 30 mL. Care is taken to avoid contaminants such as feces, menstrual blood, vaginal secretions, and prostatic fluids from mixing with the specimen. After collection, the urine was rapidly centrifuged for 20 min (1,000 g, 4°C) to remove the sediment. After centrifugation, the supernatant urine was dispensed into 1.5 mL EP tubes and continued to the next step of the experiment or frozen at −80°C. The urinary protein digestion, mass spectrometric analysis, and spectral establishment used in this study were described in our previous report (23). Urinary peptide analysis was analyzed by LC–MS/MS and performed using an Orbitrap Fusion Lumos mass spectrometer (Thermo Fisher Scientific, Waltham, MA, United States) (23). The urine proteins were expressed by normalization of the abundance of one protein/all proteins in each patient.

### The screening process and enrichment analysis of differential expression proteins

We define log2 (fold change) absolute values >1.5 and *p* values <0.05 (in MN vs. HC and MN vs. IgAN, respectively) to identify differential expression proteins (DEPs). Subsequently, all identified DEPs underwent Gene Ontology (GO) and Kyoto Encyclopedia of Genes and Genomes (KEGG) analyses by using the DAVID database.^1^ Then, the DEPs of complement components were further analyzed. And their correlations with clinicopathological features and clinical remission were investigated.

### Clinical and pathological data collection

The baseline clinical parameters, laboratory data, and renal pathological parameters of all patients while receiving renal biopsy were collected. We also collect treatment regimens and follow-up data from all patients.

According to the 2012 KDIGO guidelines, complete remission, partial remission, and no remission are defined as follows: (1) Complete remission (CR): urinary protein excretion <0.3 g/d, accompanied by normalization of serum albumin concentration and normalization of serum creatinine. (2) Partial remission (PR): urinary protein excretion <3.5 g/d, 50% or more reduction from peak, accompanied by improvement or normalization of serum albumin concentration and stable serum creatinine. (3) No remission (NR): patients did not achieve clinical remission after treatment (24).

Definitions of the various pathologic stages of MN. Stage I: normal glomerular capillaries under light microscopy, and scattered or regularly distributed small deposits of immune complex-like electron-dense material in the region of the interstitial space between the glomerular basement membrane and epithelial cells under electron microscopy. Stage II: uneven thickening of glomerular capillaries to form “spikes” under light microscopy. Electron microscopy reveals the presence of electron dense material deposits under the epithelial cells. Stage III: light microscopy shows newly formed basement membrane-like material encircling the deposits, forming a reticular or chain-like appearance. Electron dense material is deposited within the basement membrane or under the epithelium under electron microscopy. Stage IV: significant thickening of the basement membrane under light microscopy.

### Validation by enzyme-linked immunosorbent assay

We selected 11 differential complement-related proteins for further validation assay in urine samples by using ELISA kits manufactured by Shanghai Zhuo Cai, Inc. The criteria for screening urinary complement proteins for validation are as follows: (1) There should be a significant correlation with clinical or pathological parameters observed in a discovery cohort of patients with MN; (2) The proteins could represent distinct complement activation pathways; (3) Previous studies have confirmed their important role in MN; or, (4) There are complement inhibitors/antibodies available for utility. The 11 complement-related proteins were C4a, C9, CD59, CFB, CFH, CFD, MASP2, VTN, CLU, serpinc1, and colec12. All assays were conducted according to the manufacturer’s instructions. The frozen samples were rapidly thawed at 37°C and promptly transferred to the ice to prevent complement activation before dilution. After dilution, the samples were promptly loaded into microanalytical wells. Each urine sample underwent only one freeze–thaw cycle before analysis. The concentration of urinary complement analyzed by ELISA was normalized to the urinary protein-to-creatinine ratio.

### Immunohistochemistry and immunofluorescence in renal tissue

The kidney tissue embedded in paraffin was sliced into sections with a thickness of 2 μm. After deparaffinization and hydration, autoclaved antigen repair was performed. Endogenous peroxidase was blocked with 3% peroxide-methanol and incubated with goat serum working solution for 15 min at room temperature. For immunohistochemistry, primary anti-CD59 antibody at a dilution of 1:200 and primary anti-C5b-9 antibody at a dilution of 1:500 was added and incubated overnight at 4°C, followed by incubation with secondary antibodies at 37°C for 1 h. Diaminobenzidine was then added, ensuring that each sample was color developed for 3 min. Finally, the nuclei were stained with hematoxylin for 10 min. For immunofluorescence, primary anti-CD59 or anti-C5b-9 antibody diluted 1:100 was added first and incubated overnight at room temperature, followed by incubation with the corresponding fluorescein isothiophosphate (FITC-)-coupled secondary antibodies for 1 h at 37°C, and finally staining of nuclei with DAPI for 5 min. Primary anti-CD59 antibody, goat anti-mouse IgG H&L (FITC), and goat anti-rabbit IgG H&L (TRITC) were obtained from Zenbio under item numbers 220,768, 511,101 and 511,202, respectively. Primary anti-C5b-9 antibody was obtained from Abcam under item number ab55811.

### Statistical analysis

The correlations between urinary complement levels and other variables were analyzed by using correlation analysis with Spearman. Student’s *t*-test and non-parametric tests were used to assess differences between groups. Statistical significance was determined at a two-tailed *p* value (*p* < 0.05). All statistical analyses were conducted using SPSS Statistics version 26.0 (SPSS Inc., Chicago, IL, United States).

## Results

### Comparison of baseline characteristics of MN patients in the discovery and validation cohorts

There were 50 patients with MN in the discovery cohort and 80 patients in the validation cohort. There were no statistically significant differences in age, gender, 24 h proteinuria, eGFR, serum albumin, monocyte infiltration, and tubular atrophy/interstitial fibrosis (TIF) between the two cohorts. The validation cohort showed a notably higher serum creatinine level [74.40 (61.60, 90.20) μmol/L vs. 65.30 (53.40, 75.30) μmol/L, *p* = 0.007] and proportion of hypertension [39 (49%) vs. 13 (26%)] and lower serum C3 level [1.18 (0.97, 1.32) g/L vs. 1.06 (0.95, 1.19) g/L, *p* = 0.037] than the discovery cohort. However, there was no statistically significant difference in eGFR and renal C3 deposition levels between the two groups. Although the validation cohort had a significantly higher proportion of patients with hypertension than the discovery cohort, none of the hypertensive patients had hypertensive nephropathy (Table 1).

**Table 1:** Baseline demographic, clinical, and pathological characteristics of patients with MN.

Characteristics	Discovery cohort (*n* = 50)	Validation cohort (*n* = 80)	*p* value
Gender (male, %)	25 (50%)^a^	51 (64%)	0.123
Age (year)	50.7 ± 13.0^b^	51.5 (35.5, 59.0)	0.620
Hypertension (mmHg)	13 (26%)	39 (49%)	0.020
Serum creatinine (μmol/L)	65.30 (53.40, 75.30)	74.40 (61.60, 90.20)	0.007
eGFR (mL/min/1.73 m2)	100.04 ± 20.94	98.60 (82.04, 108.50)	0.055
Proteinuria (g/24 h)	3.97 (2.09, 7.71)^c^	5.29 (2.39, 8.04)	0.409
Serum albumin (g/L)	26.01 ± 6.80	26.19 ± 6.38	0.884
Serum C3 (g/L)	1.18 (0.97, 1.32)	1.06 (0.95, 1.19)	0.037
Serum C4 (g/L)	0.24 (0.20, 0.32)	0.29 ± 0.09	0.123
Basement membrane thickening			
Percentage of all patients	49 (98%)	77 (96%)	0.576
Monocyte infiltration			
Percentage of all patients	28 (56%)	55 (69%)	0.118
Per patient (%)	5 (0, 5)	5 (0, 5)	
Tubular atrophy/interstitial fibrosis			
Percentage of all patients	27 (54%)	52 (65%)	0.137
Per patient (%)	5 (0, 5)	5 (0, 5)	

MN, membranous nephropathy; eGFR, estimated glomerular filtration rate.

a Data are expressed as frequency and ratio.

b Data are expressed as means ± standard deviations.

c Data are expressed as medians and interquartile ranges (IQRs).

A total of 85 patients with MN were included in the follow-up study. This study comprised two groups: the discovery cohort, which consisted of 24 patients, and the validation cohort, which included 61 patients. Statistical analysis revealed no significant differences in baseline eGFR, proteinuria, tubular atrophy/interstitial fibrosis, or lymphocytic infiltration between the two cohorts. However, the validation cohort exhibited a higher serum creatinine level (61.7 (46.55, 74.2) μmol/L vs. 74.05 (61.08, 87.80) μmol/L, *p* = 0.014) (Supplementary Table S1).

### GO and KEGG analysis of urinary differential expression proteins

**Figure 1:**
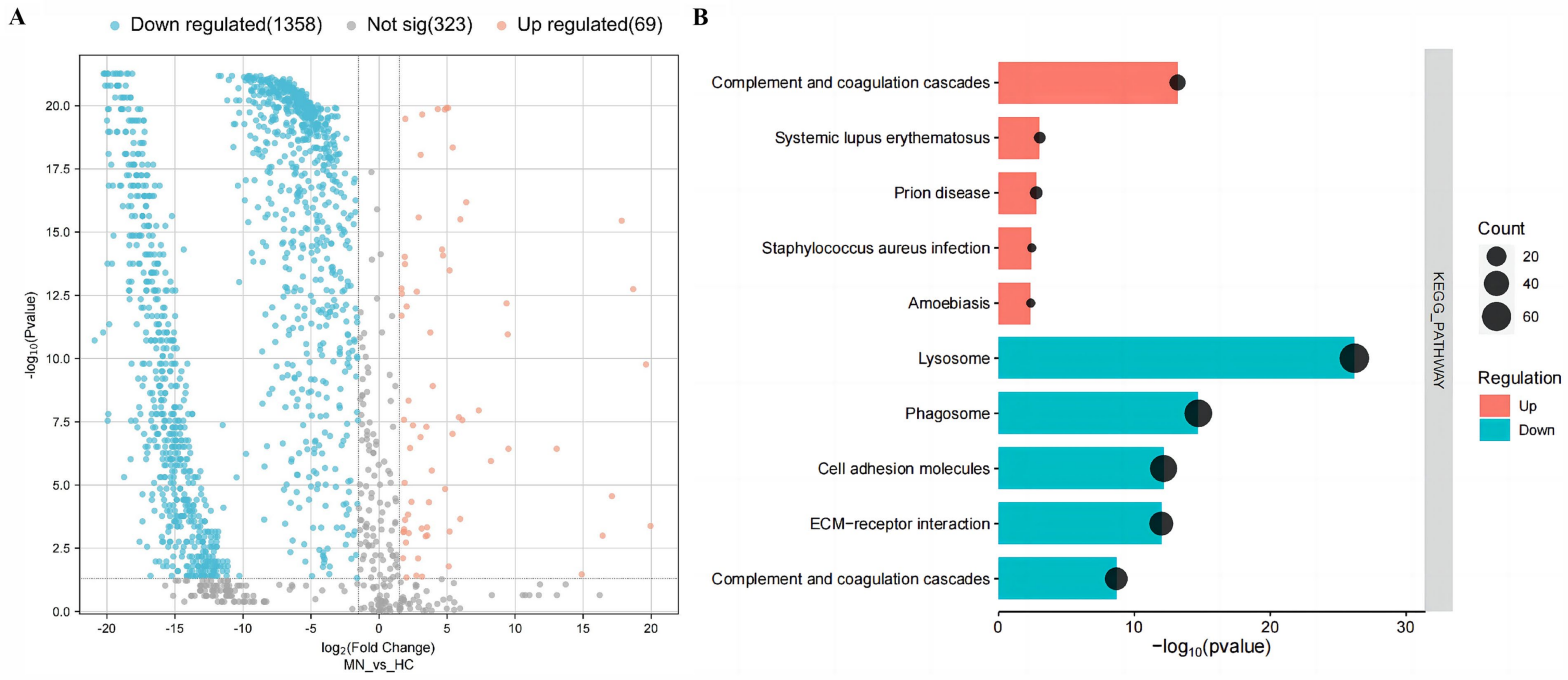
Urinary differential expression proteins and their enrichment pathways in the membranous nephropathy (MN) and healthy controls (HC). **(A)** Volcano plot of urinary differential expression proteins in the membranous nephropathy (MN) and healthy controls (HC). **(B)** KEGG-enriched dot plot of urinary differential expression proteins in MN and HC.

A total of 1750 proteins were identified in the urine of patients with MN, IgAN, and HC using LC–MS/MS. Notably, the abundance levels of 1,427 proteins exhibited significant differences between the MN and HC groups. Specifically, 69 proteins demonstrated elevated levels, whereas 1,358 proteins were found to have reduced levels (Figure 1).

To gain deep insights into the characteristics of urinary proteins in MN patients, the 1,427 DEPs were subjected to Gene Ontology (GO) analysis. In the biological process (BP) category, the DEPs were predominantly associated with mechanisms of cell adhesion and proteolysis. In the cellular component (CC) category, these proteins were primarily linked to the extracellular exosome and extracellular region. Furthermore, within the molecular function (MF) category, the proteins were chiefly associated with calcium ion binding and protein binding activities. Additionally, the KEGG enrichment analysis indicated that the up-regulated DEPs were predominantly associated with the complement and coagulation cascades. In contrast, the down-regulated DEPs were primarily linked to lysosomal pathways, as well as the complement and coagulation cascades (Figure 1) (Supplementary Figure S1).

### Correlation analysis of urinary complement proteins and both clinical and pathological features in MN patients

**Figure 2:**
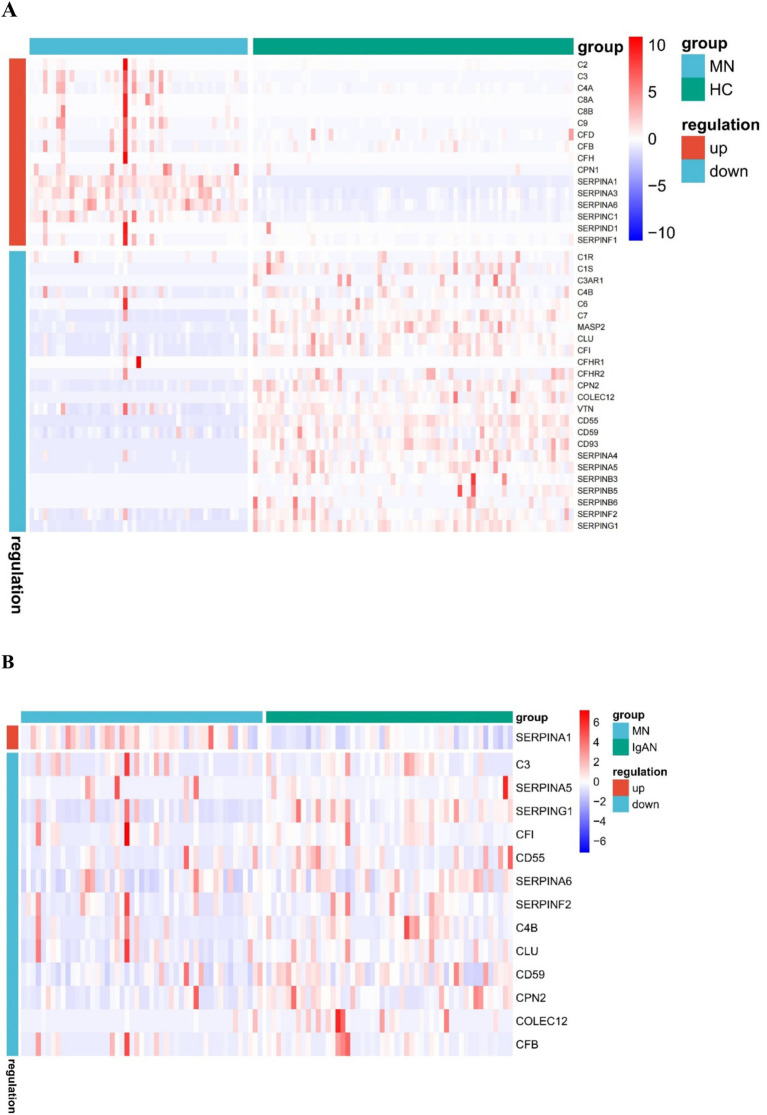
Heat map of urinary differential expression complement proteins between membranous nephropathy (MN) patients and healthy controls (HC) **(A)** MN and IgA nephropathy (IgAN) patients **(B)**.

Based on the KEGG analysis, the results prompted a focused examination of complement-related proteins. Among the quantified proteins, we identified a total of 44 complement components. Table 2 presents the comparative analysis of urinary complement proteins across the groups of MN, IgAN, and HC. The results revealed 40 complement proteins with differential expression between the MN and HC groups, of which 16 were up-regulated and 24 down-regulated. Furthermore, a comparison between patients with MN and those with IgAN identified 14 complement proteins that exhibited significant differences in abundance (*p* < 0.05), with 1 protein being up-regulated and 13 down-regulated (Figure 2).

**Table 2:** Comparisons of urinary complement proteins between MN patients and HC or IgAN patients, respectively.

Urine complements	*p* value	*p* value	Urine complements	*p* value	*p* value
	MN vs. HC	MN vs. IgAN		MN vs. HC	MN vs. IgAN
COLEC12	2.90 × 10^−20^	0.002	CPN1	2.67 × 10^−6^	0.785
C1R	0.001	0.992	CPN2	1.03 × 10^−17^	2.84 × 10^−4^
C1S	3.26 × 10^−13^	0.564	CLU	2.86 × 10^−16^	0.001
C2	0.008	0.066	VTN	8.55 × 10^−11^	0.123
C4A	0.038	0.053	CFHR1	1.26 × 10^−16^	0.529
C4B	7.53 × 10^−8^	6.59 × 10^−5^	CFHR2	1.86 × 10^−5^	0.326
MASP2	9.79 × 10^−17^	0.148	CFHR3	0.157	0.322
CFD	4.98 × 10^−5^	0.661	C3AR1	7.00 × 10^−3^	0.322
CFB	0.006	0.039	SERPINF1	0.001	0.053
CFI	9.98 × 10^−16^	0.004	SERPINC1	2.62 × 10^−16^	0.85
CFH	1.48 × 10^−4^	0.058	SERPINA1	1.23 × 10^−20^	1.80 × 10^−5^
C3	4.79 × 10^−5^	0.041	SERPINA3	9.49E × 10^−15^	0.944
C5	0.877	0.827	SERPINA5	8.05 × 10^−21^	0.001
C6	2.27 × 10^−10^	0.488	SERPING1	8.27 × 10^−20^	1.61 × 10^−8^
C7	7.90 × 10^−19^	0.156	SERPINB3	2.61 × 10^−16^	0.175
C8A	0.001	0.416	SERPINA4	8.67 × 10^−18^	0.085
C8B	2.76 × 10^−5^	0.303	SERPINF2	2.31 × 10^−12^	0.03
C8G	0.591	0.069	SERPINA6	8.72 × 10^−13^	0.015
C9	0.001	0.833	SERPINA7	0.941	0.933
CD55	3.08 × 10^−20^	0.006	SERPIND1	4.41 × 10^−10^	0.067
CD59	1.49 × 10^−12^	0.008	SERPINB6	1.04 × 10^−4^	0.322
CD93	2.15 × 10^−18^	0.159	SERPINB5	9.56 × 10^−8^	0.159

MN, membranous nephropathy; IgAN, immunoglobulin A nephropathy; HC, health controls.

**Figure 3:**
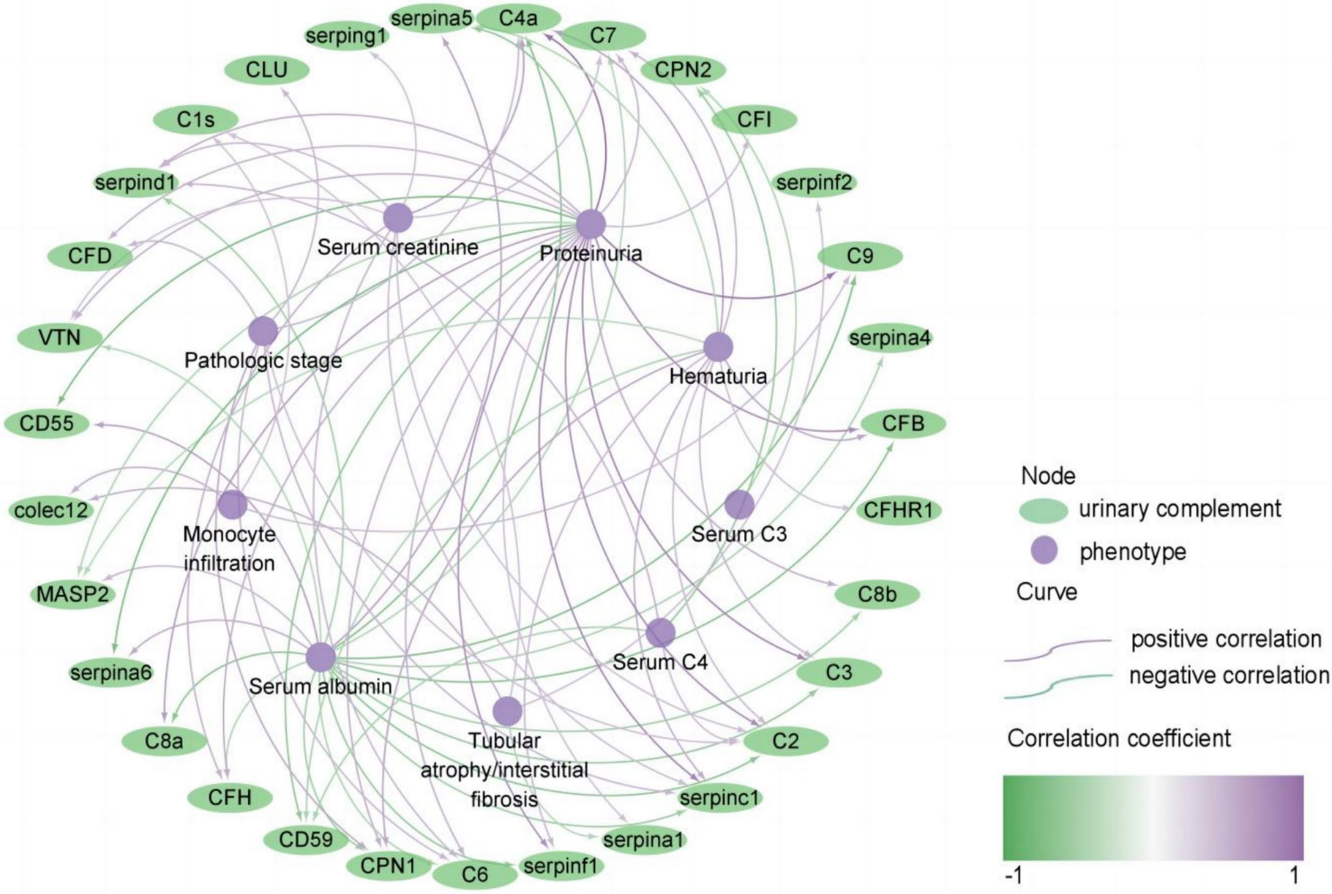
Correlation of urinary complement proteins with clinical and pathological features in patients with membranous nephropathy (MN) (only curves with *p*-values <0.05 are shown). The purple curves represent a positive correlation and the green curves represent a negative correlation, the darker the color, the stronger the correlation. The purple nodes represent phenotypes of MN, and the green nodes represent urinary complement proteins.

Then we conducted a comprehensive analysis to investigate the correlation between urinary complement proteins and a range of clinical and pathological parameters. Our results indicate a significant positive correlation between proteinuria and the levels of 21 urinary complement proteins. Notably, we observed strong associations with C4a (*r* = 0.712, *p* < 0.05), C3 (*r* = 0.615, *p* < 0.05), and C9 (*r* = 0.685, *p* < 0.05). In contrast, we identified a significant negative correlation between 24 h proteinuria and the abundance of six urinary complement proteins, including CD55 (*r* = −0.543, *p* < 0.05) and CD59 (*r* = −0.451, *p* < 0.05). We also observed a notable positive correlation between serum albumin levels and the abundance of four urinary complement proteins, specifically CD55 (*r* = 0.465, *p* < 0.05). Conversely, we identified a significant negative correlation with the abundance of 18 urinary complement proteins, including C9 (*r* = −0.520, *p* < 0.05), C4a (*r* = −0.503, *p* < 0.05), and complement factor B (CFB) (*r* = −0.493, *p* < 0.05). Serum creatinine (Scr) demonstrated a significant positive correlation with nine urinary complement proteins, notably C4a (*r* = 0.451, *p* < 0.05) and complement factor H (CFH) (*r* = 0.362, *p* < 0.05) (Figure 3) (Supplementary Table S2).

Additionally, there was a positive correlation found between the pathologic stage and the abundance of 7 urinary complement-associated proteins, including CPN1 (*r* = 0.419, *p* < 0.05), CFD (*r* = 0.345, *p* < 0.05), and serpinc1 (*r* = 0.358, *p* < 0.05). Renal tubular atrophy/interstitial fibrosis (TIF) and monocyte infiltration were positively correlated with the abundance of colec12 (*r* = 0.384, *p* < 0.05; *r* = 0.373, *p* < 0.05) and C1s (*r* = 0.292, *p* < 0.05; *r* = 0.289, *p* < 0.05) (Figure 3) (Supplementary Table S2). These findings underscore the potential role of urinary complement proteins as biomarkers for assessing proteinuria and related renal pathologies.

### Analysis of differences in urinary complement activation at different stages of progression and levels of damage

We also compared the differences in complement activation between stage II and stage I of MN. Several urinary complements were higher in patients with stage II MN than in stage I, including CFD, CLU, C6, and C4a (Supplementary Table S3). Urinary MASP2 and COLEC12 were higher in patients with tubular atrophy/interstitial fibrosis than in those without (Supplementary Table S4). Urinary CD55 was significantly higher in patients without renal tissue C3 deposition than in those with deposition (Supplementary Table S5).

### Analysis of urinary complement proteins and clinical remission of MN

In the discovery cohort, a total of 24 out of 50 patients with MN were followed up. Among these, 19 cases (79.2%) received immunosuppressive therapy (combination of steroids and cyclophosphamide; and calcineurin inhibitors, either alone or in conjunction with steroids). Meanwhile, 5 cases were managed with non-immunosuppressive supportive treatment [angiotensin-converting enzyme inhibitors (ACEI) or angiotensin II receptor blockers (ARB)].

We conducted an analysis of the relationship between clinical remission and urinary complements in patients undergoing treatment with immunosuppressants. After 3 months of immunosuppressive therapy, 10 patients (52.6%) achieved clinical remission, while 8 patients (42.1%) did not reach remission, and 1 patient (5.3%) was lost to follow-up before reaching remission. The ratio of CD55/total protein (1.10 × 10^−4^ ± 1.12 × 10^−4^ vs. 0, *p* = 0.006) and CD59/total protein [5.27 × 10^−4^(4.43 × 10^−4^, 8.09 × 10^−4^) vs. 2.05×10^−4^(2.63×10^−5^, 4.72×10^−4^), *p* = 0.012] were significantly higher in the group with clinical remission than in those without remission. While there were no statistically significant differences in the other complement components between the clinical remission and non-remission groups (Table 3).

**Table 3:** Comparisons of urinary complement protein levels and clinical parameters between the group with no remission and the group with clinical remission in the discovery cohorts after immunosuppressive therapy.

Characteristics	In 3 months of therapy	No remission (*n* = 8)	*p* value	In 6 months of therapy	No remission (*n* = 2)	*p* value
	Clinical remission (*n* = 10)			Clinical remission (*n* = 16)		
Age, years	48 ± 11^a^	53 ± 17	0.501	49 ± 14	58 ± 8	0.402
Serum creatinine (μmol/L)	65.07 ± 18.06	67.40 (48.23, 75.55)	0.829	61.70 (47.88, 76.30)	72.90 ± 4.43	0.47
eGFR (mL/min/1.73 m2)	107.11 (99.77, 115.23)^b^	96.49 ± 22.37	0.203	104.46 (94.95, 113.35)	97.46 ± 8.21	0.549
Proteinuria (g/24 h)	3.73 (2.04, 6.38)	7.07 ± 5.58	0.408	4.18 (2.26, 7.68)	11.39 ± 11.07	0.392
Serum albumin (g/L)	25.5 ± 5.77	25.15 ± 8.09	0.916	25.16 ± 6.21	26.85 ± 13.22	0.746
CD55/all protein	1.10 × 10^−4^ ± 1.12 × 10^−4^	0 (0, 0)	0.006	2.50 × 10^−4^ (1.12 × 10^−6^, 1.51 × 10^−4^)	0 ± 0	0.118
CD59/all protein	5.27 × 10^−4^ (4.43 × 10^−4^, 8.09 × 10^−4^)	2.05×10^−4^ (2.63×10^−5^, 4.72×10^−4^)	0.012	5.07 × 10^−4^ (3.13 × 10^−4^, 6.31 × 10^−4^)	0 ± 0	0.013
C3/all protein	8.52 × 10^−4^ (2.06 × 10^−4^, 2.29 × 10^−3^)	4.56 × 10^−3^ (3.62 × 10^−4^, 1.55 × 10^−2^)	0.173	2.40 × 10^−4^ (0, 6.51 × 10^−4^)	1.22 × 10^−2^ ± 5.38 × 10^−3^	0.078
C9/all protein	1.38 × 10^−4^ (5.74 × 10^−5^, 2.50 × 10^−4^)	5.37 × 10^−4^ (2.13 × 10^−5^, 1.58 × 10^−3^)	0.46	6.29 × 10^−5^ (0, 1.48 × 10^−4^)	3.55 × 10^−4^ ± 5.02 × 10^−4^	0.732
CFB/all protein	6.37 × 10^−5^ (1.28 × 10^−5^, 2.76 × 10^−4^)	3.58 × 10^−4^ (9.14 × 10^−6^, 1.05 × 10^−4^)	0.633	1.23 × 10^−5^ (0, 6.54 × 10^−5^)	1.39 × 10^−3^ ± 1.96 × 10^−3^	1
CFH/all protein	1.77 × 10^−6^ (0, 1.73 × 10^−5^)	0 (0, 4.43 × 10^−5^)	0.897	0 (0, 2.71 × 10^−5^)	3.40 × 10^−6^ ± 4.81 × 10^−6^	0.941
CLU/all protein	4.23 × 10^−4^ (1.70 × 10^−4^, 6.27 × 10^−4^)	6.84 × 10^−4^ (2.03 × 10^−4^, 1.46 × 10^−3^)	0.408	2.03 × 10^−4^ (0, 4.42 × 10^−4^)	2.29 × 10^−3^ ± 3.24 × 10^−3^	1
MASP2/all protein	1.72 × 10^−4^ (4.77 × 10^−5^, 5.89 × 10^−4^)	1.26 × 10^−4^ (4.62 × 10^−6^, 9.64 × 10^−4^)	0.633	1.39 × 10^−4^ (4.38 × 10^−5^, 5.16 × 10^−4^)	6.25 × 10^−4^ ± 8.58 × 10^−4^	0.941

a Data are expressed as means ± standard deviations.

b Data are expressed as medians and interquartile ranges (IQRs).

After 6 months of immunosuppressive therapy, 16 (84.2%) achieved clinical remission, 2 (10.5%) did not reach remission, and 1 (5.3%) was lost to follow-up. Only the ratio of CD59/all protein [5.07 × 10^−4^ (3.13 × 10^−4^, 6.31 × 10^−4^) vs. 0, *p* = 0.013] were significantly higher in the group with clinical remission than in the group with no remission (Table 3).

### Correlation between urinary complement proteins and clinical and pathologic parameters in the validation cohort of MN patients

A total of 11 complement proteins, including Colec12, MASP2, CFB, CFH, CFD, C4A, C9, VTN, CLU, and Serpinc1, were further validated in a cohort of 80 patients with MN using ELISA. Our results revealed that most urine complement to urine creatinine ratios exhibited a negative correlation with serum uric acid levels, which aligns with the findings from the discovery cohort. This may be due to the fact that the complement and coagulation cascade pathways are major functional pathways involved in the development of gout in hyperuricemic patients. Additionally, we observed no significant correlation between urinary complement proteins and the levels of anti-phospholipase A2 receptor antibody IgG (anti-PLA2R-IgG). But we did not find a strong correlation between urinary complements and pathologic parameters in the validation cohort (Table 4).

**Table 4:** Correlation analysis of 11 urinary complement proteins with clinical and pathological parameters in patients with MN.

Characteristics	CLU/Cr	CFB/Cr	C9/Cr	MASP2/Cr	C4a/Cr	colec12/Cr	VTN/Cr	Serpinc1/Cr	CFD/Cr	CFH/Cr	CD59/Cr
Serum creatinine (μmol/l)	*r* = −0.167	*r* = −0.314^*^	*r* = −0.191	*r* = −0.245^*^	*r* = −0.211	*r* = −0.22	*r* = −0.204	*r* = −0.185	*r* = −0.239^*^	*r* = −0.249^*^	*r* = −0.211
Serum uric acid (mmol/l)	*r* = −0.375^*^	*r* = −0.305^*^	*r* = −0.324^*^	*r* = −0.297^*^	*r* = −0.334^*^	*r* = −0.328^*^	*r* = −0.411^*^	*r* = −0.282^*^	*r* = −0.299^*^	*r* = −0.307^*^	*r* = −0.278^*^
Serum Albumin (g/L)	*r* = 0.014	*r* = 0.108	*r* = 0.042	*r* = 0.024	*r* = −0.022	*r* = −0.022	*r* = −0.002	*r* = −0.122	*r* = 0.008	*r* = −0.017	*r* = −0.042
Serum C3 (g/L)	*r* = 0.021	*r* = −0.054	*r* = 0.034	*r* = 0.014	*r* = −0.047	*r* = −0.008	*r* = 0.013	*r* = 0.052	*r* = 0.047	*r* = 0.108	*r* = 0.018
Proteinuria (g/24 h)	*r* = −0.152	*r* = −0.248^*^	*r* = −0.156	*r* = −0.139	*r* = −0.122	*r* = −0.129	*r* = −0.166	*r* = −0.073	*r* = −0.157	*r* = −0.117	*r* = −0.08
anti-PLA2R-IgG (RU/ml)	*r* = 0.086	*r* = −0.247	*r* = 0.096	*r* = −0.021	*r* = 0.093	*r* = 0.13	*r* = 0.049	*r* = −0.008	*r* = 0.066	*r* = 0.053	*r* = 0.012
TIF (%)	*r* = 0.022	*r* = −0.04	*r* = −0.003	*r* = −0.016	*r* = −0.021	*r* = −0.016	*r* = 0.026	*r* = 0.063	*r* = 0.001	*r* = −0.01	*r* = −0.01
Monocyte infiltration (%)	*r* = 0.16	*r* = 0.062	*r* = 0.151	*r* = 0.079	*r* = 0.067	*r* = 0.127	*r* = 0.188	*r* = 0.222	*r* = 0.093	*r* = 0.09	*r* = 0.13
Pathological staging	*r* = 0.005	*r* = −0.026	*r* = 0.012	*r* = −0.044	*r* = −0.072	*r* = 0.004	*r* = −0.027	*r* = −0.059	*r* = −0.034	*r* = −0.106	*r* = −0.041

TIF, tubular atrophy/interstitial fibrosis; MN, membranous nephropathy; anti-PLA2R-IgG, anti-phospholipase A2 receptor antibody IgG; ^*^ *p* < 0.05.

### Analysis of urinary complement proteins and clinical remission of MN in the validation cohort

In the validation cohort, out of the 80 patients with MN, 9 were excluded due to lack of follow-up records and 10 for lacking of proteinuria, and a total of 61 patients with MN were included for further analysis. 58 cases (95.1%) received immunosuppressive therapy (including corticosteroids, rituximab) and 3 cases (4.9%) received supportive care. Undergoing 3 months of immunosuppressive therapy, 20 (34.5%) achieved clinical remission, 29 (50%) were not in remission, and 9 (15.5%) were lost to follow-up. There were no significant differences in complement levels and clinical parameters between the clinical remission and non-remission groups between the two groups. After 6 months of immunosuppressive therapy, 33 (56.9%) achieved clinical remission, 13 (22.4%) were not in remission, and 12 (20.7%) were lost to follow-up. The ratio of CD59/Cr (147.73 (84.45, 300.18) ng/mg, vs. 63.45 (499.18, 136.95) ng/mg, *p* = 0.021) were significantly higher in the group with clinical remission than in the group with no remission (Table 5).

**Table 5:** Comparisons of urinary complement protein levels and clinical parameters between the group with no remission and the group with clinical remission in the validation cohorts after immunosuppressive therapy.

Characteristics	In 3 months of therapy	No remission (*n* = 29)	*p* value	In 6 months of therapy	No remission (*n* = 13)	*p* value
	Clinical remission (*n* = 20)			Clinical remission (*n* = 33)		
Age, years	52 ± 13^a^	47 ± 17	0.366	53 (48, 62)	45 ± 17	0.143
Serum creatinine (μmol/L)	75.85 ± 19.62	76.79 ± 23.00	0.882	74.99 ± 21.63	83.44 ± 18.81	0.238
eGFR (mL/min/1.73 m2)	93.25 ± 18.05	98.84 (75.04, 115.26)	0.771	2.04 ± 19.74	93.06 ± 23.01	0.885
Proteinuria (g/24 h)	4.37 (2.65, 7.94)^b^	5.29 (2.16, 8.37)	0.07	4.14 (2.39, 7.00)	8.44 ± 5.37	0.07
Serum albumin (g/L)	26.02 ± 5.84	24.20 (19.50, 27.10)	0.207	26.50 ± 5.67	24.00 (18.68, 27.00)	0.207
CD59/Cr (ng/mg)	114.34 (56.20, 270.76)	101.51 (52.70, 276.30)	0.984	147.73 (84.45, 300.18)	63.45 (499.18, 136.95)	0.021

a Data are expressed as means ± standard deviations.

b Data are expressed as medians and interquartile ranges (IQRs).

### Immunohistochemical and immunofluorescence analysis of C5b-9 and CD59 in renal tissues

**Figure 4:**
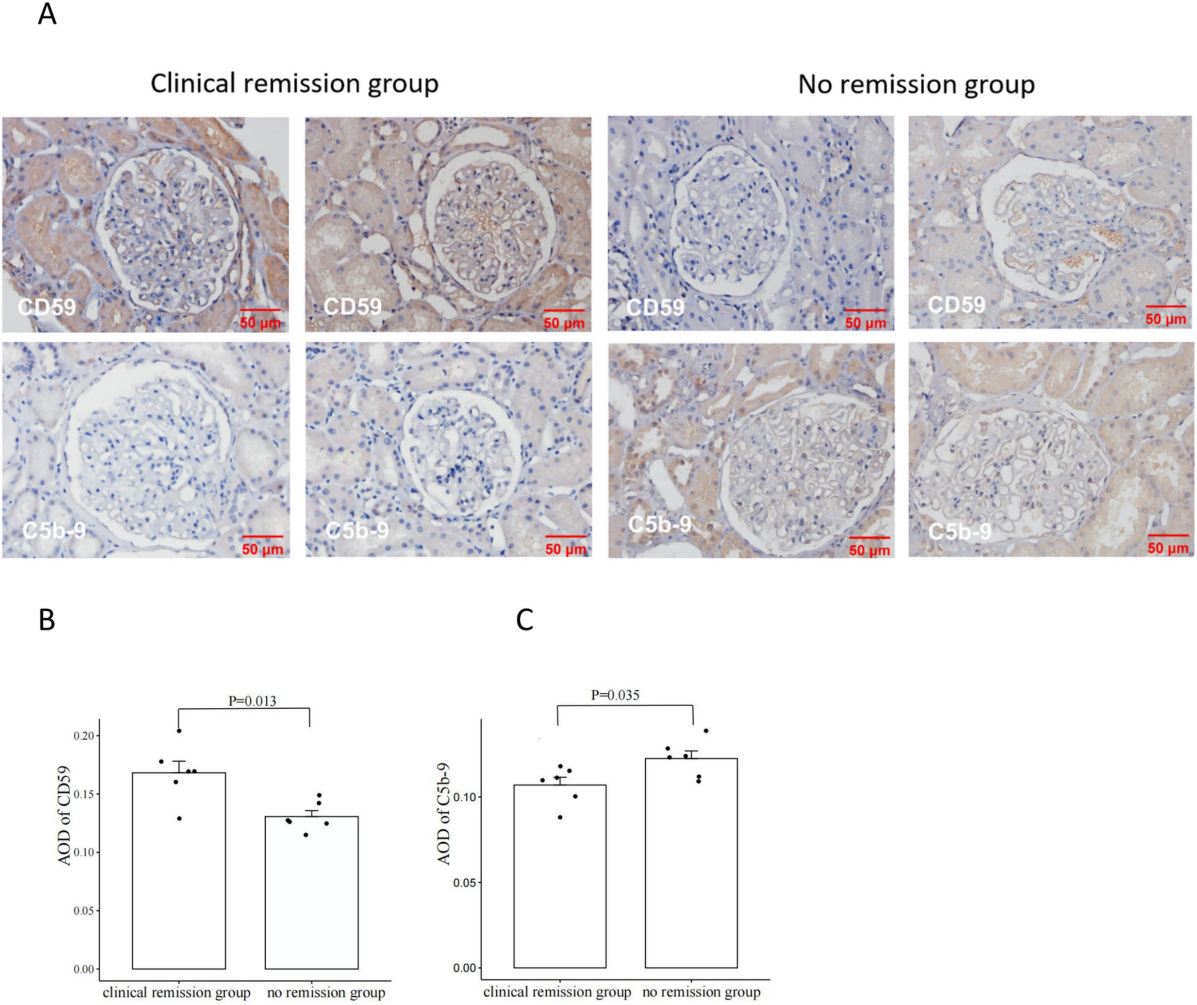
Comparison of CD59 and C5b-9 immunohistochemistry in renal tissues from patients with clinical remission versus those without remission of membranous nephropathy (MN) **(A)**. The clinical remission group had higher expression of CD59 and lower expression of C5b-9; the no remission group had lower expression of CD59 and higher expression of C5b-9. Comparison of CD59 immunohistochemistry staining intensity between the group with clinical remission and the group with no remission **(B)**. CD59 was significantly higher in the clinical remission group than in the no remission group. Comparison of C5b-9 immunohistochemistry staining intensity between the group with clinical remission and the group with no remission **(C)**. C5b-9 was significantly lower in the clinical remission group than in the no remission group. The staining intensity was measured by average OD (AOD).

**Figure 5:**
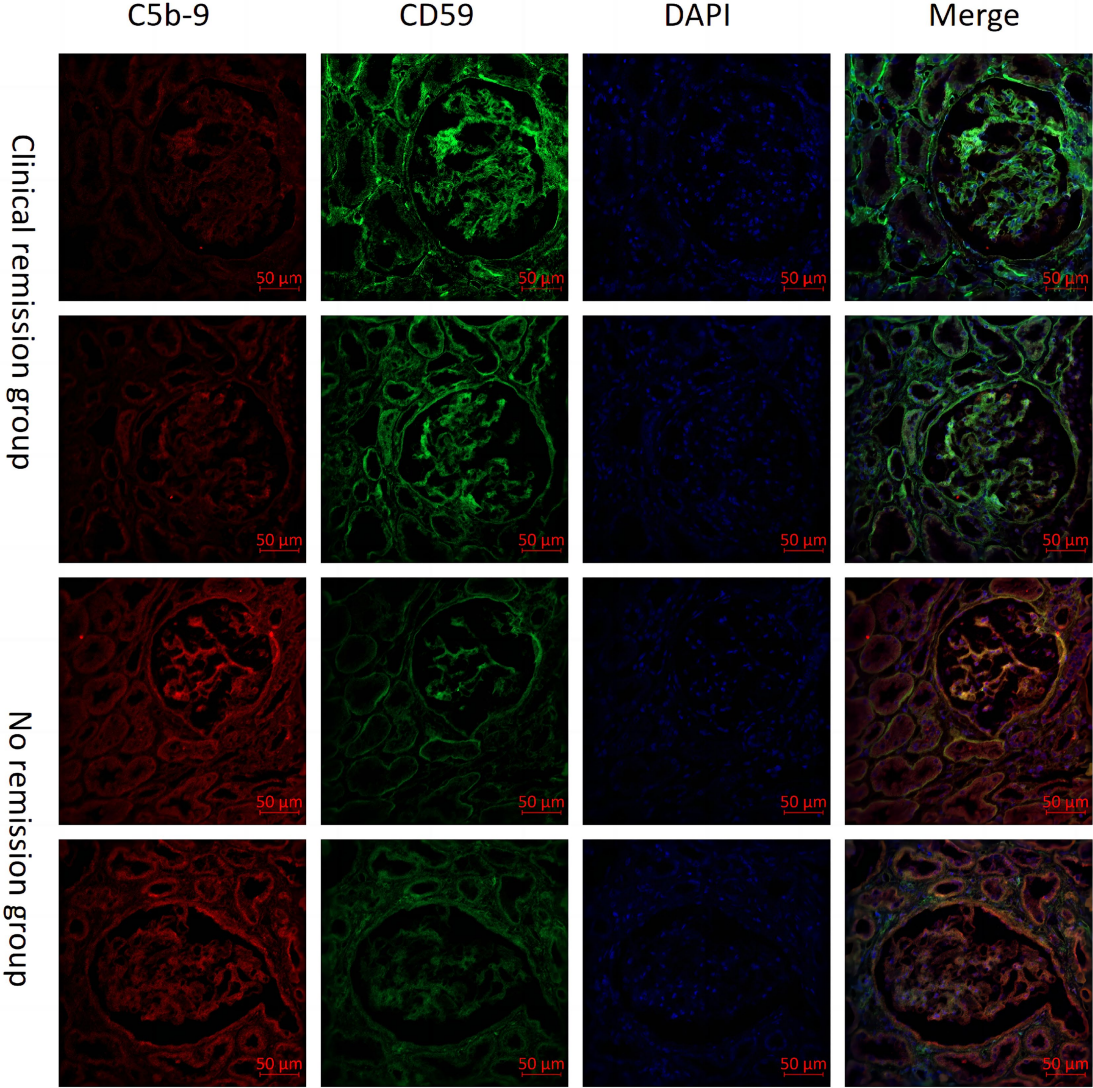
Comparison of CD59 and C5b-9 immunofluorescence immunohistochemistry in renal tissues from patients with clinical remission versus those without remission of membranous nephropathy (MN).

We also examined the expression of CD59 and C5b-9 (which was not detected by the urine proteomics assay) in renal tissue. Our study included 6 patients with MN who achieved clinical remission after 3 months of immunosuppressive therapy, as well as 6 patients who did not achieve remission despite undergoing 6 months of treatment. We observed higher expression of CD59 and lower expression of C5b-9 in the clinical remission group, showing the opposite expression trend in the no remission group. Then we performed a quantitative analysis and found statistically significant differences in CD59 and C5b-9 between the two groups (Figure 4). Additionally, immunofluorescence analyses confirmed these findings, showing the localization of C5b-9 and CD59 in the glomeruli and tubules (Figure 5).

## Discussion

We conducted a thorough analysis of urine proteomic data to investigate the differential expression of complement-related proteins in patients with MN. Our results revealed that lots of complement components were abnormal in these patients, and we observed correlations with their clinicopathological characteristics. These findings suggest that the activation of the complement system may play a crucial role in the pathophysiological processes associated with MN.

The complement system, first identified by Jules Bordet in 1901, is regarded as one of the oldest components of the immune system (25). The system comprises nearly 60 proteins, including those involved in the classical, lectin, and alternative pathways, along with multiple regulatory factors and receptors (26, 27). Complement activation has long been recognized as a key characteristic of MN, primarily occurring through the lectin and alternative pathways, but recent studies, however, have emphasized the classical pathway as an important contributor to the its progression (28). Our results indicate that urinary complement proteins of abnormal abundance are involved in the three pathways of complement activation, with significant enrichment in the alternative and terminal pathways.

According to previous researches, serum CFH and CFB are suggested as potential indicators of disease progression (15, 16). The factor B inhibitor LNP023 and the factor D inhibitor BCX9930 are currently in phase II clinical trials for MN. Our research reveals elevated levels of CFB and CFH in urine, with both urine CFB and urine CFH showing a positive correlation with proteinuria and Scr, and negatively correlated with serum albumin. These findings further highlight the significance of CFB and CFH in chronic kidney injury and suggest their potential as biomarkers for chronic damage. Previous studies have found that the intensity of glomerular C3 staining correlates with proteinuria (4, 5) and is a predictor of renal failure in patients with MN (6). Other studies have also demonstrated an association between glomerular C5b-9 and renal dysfunction in MN (7). APL-2, a C3 inhibitor, is currently undergoing phase II clinical trials for the treatment of MN. Our findings revealed significantly elevated levels of urine C3 and C9 in MN patients compared to healthy controls, and these levels exhibited a positive correlation with proteinuria, consistent with previous studies (20–22). It suggests that end-pathway activation plays a critical role in MN. Additionally, a correlation of C1s with TIF and monocyte infiltration was observed, further supporting the potential involvement of the classical pathway in the pathogenesis of MN. However, the initiation pathways and pathogenic relevance of complement activation in MN are not fully understood. In the future, we will explore the pathways of complement activation to gain a more comprehensive understanding of complement activation in MN.

We compared glomerular complements of MN patients detected by MS/MS (11, 12) with urine complements in MN detected by LC–MS/MS. Of the twenty-nine complement proteins detected in glomeruli, 21 (72.4%) were also detectable in urine. C3 was the complement with the highest spectral number in both glomeruli and urine. Glomerular C3 deposition leads to progression of MN to renal failure (6). Urine C3 correlates positively with proteinuria and cystatin C and negatively with serum albumin. CFHR2, CFHR3, and C1S, which are less abundant in glomeruli, are also less abundant in urine. This suggests that urine complements may be a better response to complement activation in glomeruli.

In our previous works, we concentrated on examining the factors associated with the progression and clinical remission of MN (29–31). Our investigation revealed that factors such as anti-PLA2R-IgG titer, inflammatory cell infiltration, and C3 deposition on immunofluorescence were correlated with an elevated risk of treatment failure in patients with PMN (32). In this study, we included measurements of urine complements and found that in the discovery cohort, the ratio of CD59/all protein was significantly higher in the group with clinical remission than in the group with no remission at months 3 and 6 of treatment. Showing the same in the validation cohort, the CD59/Cr was significantly higher in the group with clinical remission than in the group with no remission at month 6 of treatment. However, at month 3 of treatment, although the CD59/Cr was higher in the group with clinical remission than in the group with no remission, the difference was not statistically significant. It may be due to the fact that the validation cohort had higher baseline proteinuria and poorer renal function compared to the discovery cohort, and therefore required prolonged treatment to achieve clinical remission. Thus, we found a strong association between CD59 and clinical remission.

The process of complement activation ultimately leads to the formation of membrane attack complexes (MAC), resulting in cellular destruction. CD59, also known as homology restriction factor-20 (HRF-20), serves as the sole membrane-bound inhibitor that impedes the assembly of MAC and is present in various renal cell types, including glomerular endothelium, tubular epithelium, thylakoid cells, and podocytes (33, 34). Previous animal studies have demonstrated the significant protective effects of CD59 on the kidneys (35–38). Targeting CD59 for treating tubulointerstitial injury in rats has shown promising results (39). It has been observed that CD59 expression in the glomeruli of patients with MN is decreased compared to HC, and urine CD59 excretion is increased in comparison with HC (40). Our study revealed that urine CD59 was significantly lower in MN patients compared to both HC and IgAN patients. Additionally, CD59 exhibited a negative correlation with proteinuria and was associated with the clinical remission of MN patients. The group with clinical remission exhibited elevated levels of CD59 in both renal tissue and urine, whereas patients in the group with no remission exhibited the opposite pattern. Furthermore, patients in the group with no remission had lower renal tissue CD59 and higher C5b-9 compared to patients in the group with clinical remission. Immunofluorescence co-localization indicated that CD59 and C5b-9 were co-localized in glomeruli and tubules. We hypothesized that MN patients are more susceptible to renal attack due to the deficiency of CD59 in renal tissues, which diminishes its inhibition of C5b-9. We will focus on the precise mechanisms by which these complement proteins influence disease progression and remission in MN and explore their utility in clinical practice.

The activated components in the complement system vary at different stages of progression and degrees of damage in MN. We found that urinary complement activation levels in stage II of MN were generally higher than those in stage I. There were several differentially expressed urinary complements such as CFD, CLU, C6, and C4a, which were derived from the alternative pathway, the lectin pathway, and the common pathway, respectively. Urinary MASP2, and COLEC12 were higher in patients with tubular atrophy/interstitial fibrosis than in those without. Urinary CD55 was significantly higher in patients without renal tissue C3 deposition than in those with deposition. CD55 acts as a complement inhibitory factor, inhibiting the formation and activity of C3 converting enzyme and C5 converting enzyme, and preventing overactivation of the complement system. From this, we hypothesized that high CD55 expression has a stronger inhibitory effect on the complement system, resulting in less renal C3 deposition. In summary, urinary complement activation levels vary at different stages of progression and degree of injury in MN. Whether the degree of injury and progression of MN can be inferred by monitoring urinary complement levels requires more in-depth study. We will take it into consideration seriously in the future.

Our study uses targeted mass spectrometry for comprehensive quantification of urinary complement proteins. It demonstrates the profile of urinary complement proteins in patients with MN. The relationship between urinary complement proteins and clinicopathological features and clinical remission was analyzed. However, this study has important limitations. We did not monitor the dynamics of complement changes in MN patients after different drug treatments. Longitudinal studies are needed to monitor complement activation over longer periods of time and could provide additional insights into how chronic complement activation leads to long-term kidney injury and whether it predicts relapse or remission. We did not analyze renal PLA2R and IgG1-4 expression in relation to urinary complement characteristics. The relationship between complement activation and antigenic and IgG subtypes in renal tissue needs to be explored further. Urine specimens were not collected at exactly the same time. The impact of timing on urine protein and complement activation could be explored further. In addition, the origin of the complement proteins detected in urine is unknown. We will focus on identifying the source of these proteins, whether they are secreted by the kidneys, or are being filtered from the bloodstream due to glomerular damage.

In summary, the study showed significant abnormalities in urine complement abundance in patients with MN. The urine levels of complement components exhibited a significant correlation with clinical parameters and the clinical remission of MN patients. Consequently, these components hold potential as biomarkers for predicting MN progression and clinical remission. Our study implies for the use of complement-targeted therapy in MN.

## Data Availability

The datasets presented in this study can be found in online repositories. The names of the repository and accession number(s) can be found at http://www.proteomexchange.org/ [PXD060494].

## Footnotes

^1^ https://david.ncifcrf.gov/
