# Supplementary material for: Urine complement analysis implies complement activation is involved in membranous nephropathy
Source: Front Med (Lausanne). 2025 Feb 13;12:1515928. doi: 10.3389/fmed.2025.1515928 (PMC11865186; doi:10.3389/fmed.2025.1515928)
Supplement: Supplementary file 1 [file Image_1.pdf]

Supplementary Figure 1: Gene Ontology bubble plots of urinary differential expression proteins between membranous nephropathy (MN) and healthy controls (HC). Gene Ontology (GO) bubble plots of urinary differential expression proteins in biological process (BP), cellular component (CC), and molecular function (MF) between MN and HC, respectively. (A) Urinary differential expression proteins in BP; (B) Urinary differential expression proteins in CC; (C) Urinary differential expression proteins in MF.

A

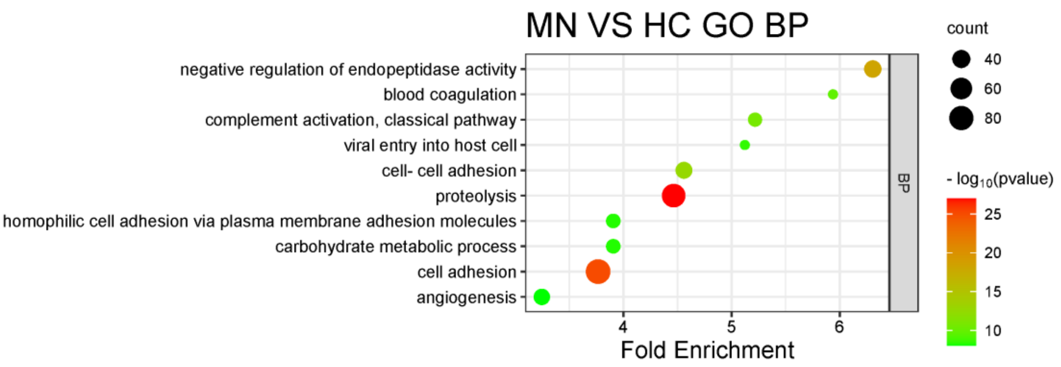

B

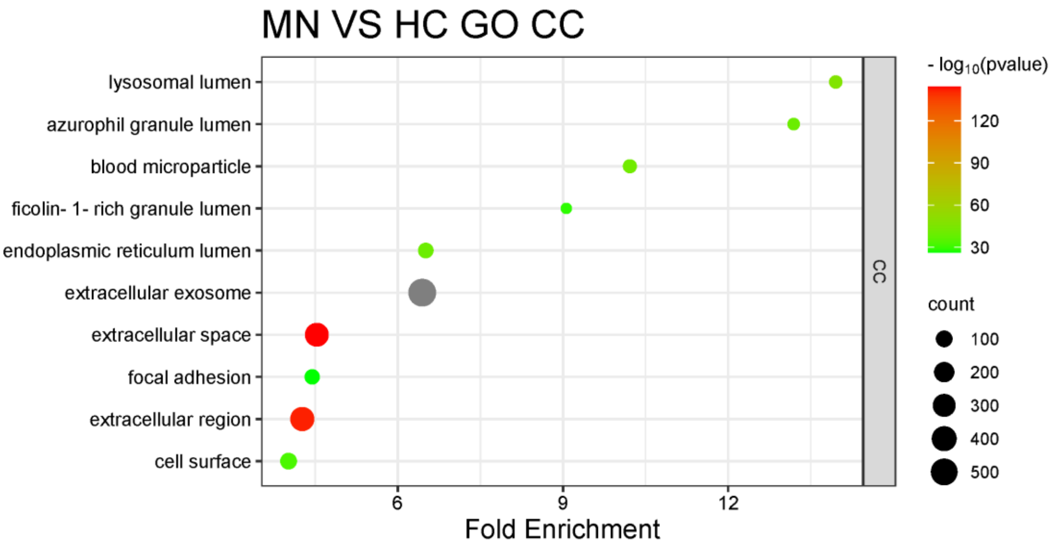

C

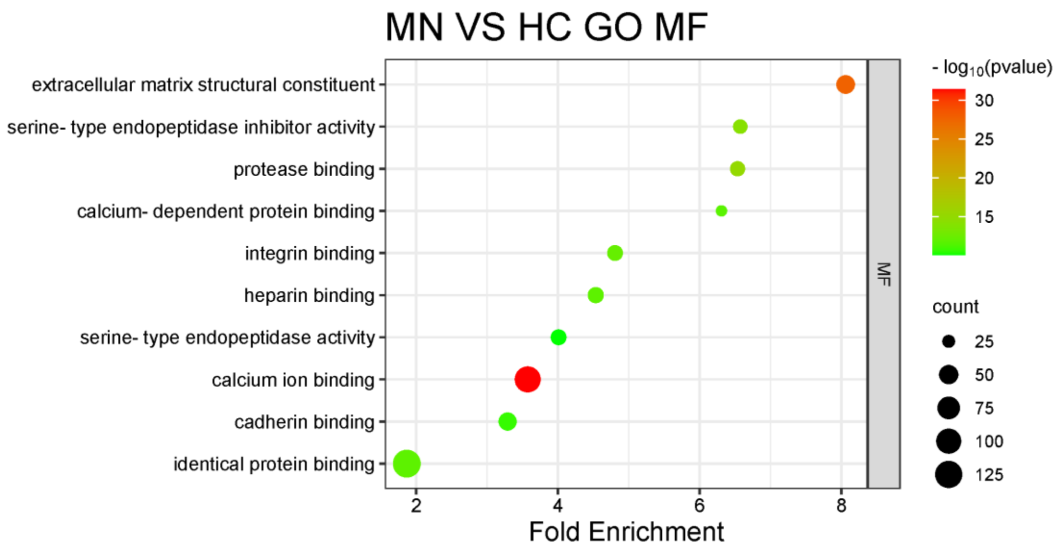
MN VS HC GO MF
extracellular matrix structural constituent
serine- type endopeptidase inhibitor activity
protease binding
calcium- dependent protein binding
integrin binding
heparin binding
serine- type endopeptidase activity
calcium ion binding
cadherin binding
identical protein binding
2
4
6
8
Fold Enrichment
MF
- log10(pvalue)
30
25
20
15
count
25
50
75
100
125
