# Supplementary material for: Urine complement analysis implies complement activation is involved in membranous nephropathy
Source: Front Med (Lausanne). 2025 Feb 13;12:1515928. doi: 10.3389/fmed.2025.1515928 (PMC11865186; doi:10.3389/fmed.2025.1515928)
Supplement: Supplementary file 2 [file Table_1.docx]

**Supplementary Table 1. Baseline demographic, clinical, and pathological characteristics of patients with MN followed up**

| Characteristics | Discovery cohort (n=24) | Validation cohort (n=61) | *P* Value |
| --- | --- | --- | --- |
| Gender (male, %) | 12 (50%)^a^ | 39 (64%) | 0.241 |
| Age (year) | 49.71 ± 12.36^b^ | 49.3 ± 16.28 | 0.911 |
| Serum creatinine (μmol/L) | 61.7 (46.55, 74.2)^c^ | 74.05 (61.08, 87.80) | 0.014 |
| eGFR (mL/min/1.73m2) | 105.65 (95.97, 114.35) | 94.75 ± 21.03 | 0.068 |
| Proteinuria (g/24h) | 3.62 (2.28, 7.35) | 4.30(2.25, 8.08) | 0.533 |
| Serum albumin (g/L) | 26.17 ± 6.52 | 26.22 ± 6.04 | 0.975 |
| Serum C3 (g/L) | 1.1 (0.94,1.29) | 1.06 ± 0.21 | 0.265 |
| Serum C4 (g/L) | 0.23 (0.20, 0.30) | 0.27 (0.22, 0.34) | 0.183 |
| Monocyte infiltration |  |  |  |
| Percentage of all patients | 13 (54%) | 41 (67%) | 0.293 |
| Per patient (%) | 5 (0, 5) | 5 (0, 5) |  |
| Tubular atrophy/interstitial fibrosis |  |  |  |
| Percentage of all patients | 10 (42%) | 39 (64%) | 0.066 |
| Per patient (%) | 0 (0, 5) | 5 (0, 5) |  |

MN, membranous nephropathy; eGFR, estimated glomerular filtration rate. ^a^: Data are expressed as frequency and ratio. ^b^: Data are expressed as means ± standard deviations. ^c^: Data are expressed as medians and interquartile ranges (IQRs).

**Supplementary Table 2. Correlation of urinary complement proteins with clinical and pathological features in patients with MN**

| **urinary complements** | **Proteinuria (g/24h)** | **Serum creatinine (μmol/l)** | **Serum Albumin (g/L)** | **Serum uric acid (mmol/l)** | **Serum C3 (g/L)** | **Serum C4 (g/L)** | **Mesangial proliferation** | **behead** | **Tubular atrophy/interstitial fibrosis** | **Monocyte infiltration** | **pathological staging** |
| --- | --- | --- | --- | --- | --- | --- | --- | --- | --- | --- | --- |
| C1R | 0.095 | -0.091 | -0.111 | -0.285 | 0.06 | -0.224 | 0.072 | 0.135 | 0.038 | 0.033 | -0.145 |
| C1S | 0.196 | 0.25 | -0.19 | -0.13 | . | . | 0.03 | -0.134 | .292^*^ | .289^*^ | 0.121 |
| C2 | .582^*^ | .304^*^ | -.477^*^ | -0.131 | 0.212 | .318^*^ | 0.085 | -0.272 | -0.119 | 0.223 | .304^*^ |
| C3 | .615^*^ | 0.254 | -.428^*^ | -0.047 | 0.087 | 0.097 | -0.122 | -0.084 | -0.095 | 0.181 | 0.105 |
| C4A | .712^*^ | .451^*^ | -.503^*^ | 0.109 | -0.014 | 0.169 | -0.02 | -.321^*^ | -0.032 | 0.233 | .338^*^ |
| C4B | 0.254 | 0.274 | -0.172 | -0.044 | 0.032 | 0.134 | -0.054 | -0.256 | 0.152 | 0.144 | 0.253 |
| C5 | .508^*^ | 0.284 | -.475^*^ | -0.089 | 0.261 | .319^*^ | 0.063 | -.285^*^ | 0.011 | 0.218 | 0.259 |
| C6 | .373^*^ | .347^*^ | -.323^*^ | -0.013 | 0.029 | 0.277 | 0.063 | -.285^*^ | 0.126 | 0.209 | .338^*^ |
| C7 | .358^*^ | .301^*^ | -.312^*^ | -0.045 | -0.033 | -0.126 | -0.021 | -0.249 | -0.088 | 0.116 | 0.266 |
| C8A | .477^*^ | 0.049 | -.472^*^ | -0.286 | 0.079 | -0.017 | -0.272 | -0.124 | -0.038 | 0.269 | 0.157 |
| C8B | .402^*^ | 0.049 | -.379^*^ | -0.247 | 0.153 | -0.021 | -0.237 | -0.121 | 0.008 | 0.282 | 0.1 |
| C8G | .476^*^ | 0.265 | -.405^*^ | -0.225 | 0.142 | 0.2 | 0.072 | -0.231 | -0.008 | 0.176 | 0.267 |
| C9 | .685^*^ | 0.228 | -.520^*^ | -0.166 | 0.204 | 0.053 | -0.031 | -0.235 | 0.148 | .319^*^ | 0.238 |
| CFD | .362^*^ | 0.256 | -0.276 | -0.09 | 0.058 | 0.024 | -0.192 | -.292^*^ | 0.09 | 0.252 | .345^*^ |
| CFB | .577^*^ | 0.129 | -.493^*^ | -0.168 | 0.125 | 0.097 | -0.093 | -0.127 | -0.05 | 0.095 | 0.171 |
| CFH | .390^*^ | .362^*^ | -.308^*^ | 0.06 | 0.016 | 0.102 | -0.183 | -0.217 | 0.129 | 0.127 | 0.256 |
| CFHR1 | 0.248 | 0.133 | -0.264 | -0.012 | 0.011 | 0.092 | 0.049 | -0.075 | 0.096 | 0.086 | 0.061 |
| CFHR2 | 0.212 | 0.081 | -0.135 | -0.155 | -0.089 | -0.124 | 0.043 | -0.031 | 0.19 | 0.181 | 0.137 |
| CFHR3 | .303^*^ | 0.237 | -0.241 | -0.22 | 0.135 | -0.038 | 0.054 | -0.242 | 0.036 | 0.254 | 0.219 |
| CFI | .323^*^ | 0.145 | -0.237 | -0.193 | 0.107 | -0.006 | -0.123 | -0.236 | 0.124 | 0.225 | 0.266 |
| MASP2 | -.317^*^ | 0.054 | .343* | 0.037 | -0.079 | -0.255 | -0.123 | -0.04 | 0.265 | 0.167 | 0.014 |
| CLU | 0.224 | 0.227 | -0.217 | -0.084 | 0.166 | 0.109 | -0.092 | -.328^*^ | 0.05 | 0.115 | .344^*^ |
| VTN | .380^*^ | .288^*^ | -.302^*^ | -0.146 | 0.046 | -0.095 | -0.123 | -0.156 | 0.041 | 0.118 | 0.173 |
| CD55 | -.543^*^ | -0.054 | .465^*^ | 0.029 | -0.255 | -0.284 | -0.042 | 0 | 0.191 | -0.046 | -0.011 |
| CD59 | -.451^*^ | -0.133 | 0.251 | -0.044 | -0.13 | -.342^*^ | -0.143 | 0.058 | 0.206 | -0.053 | -0.066 |
| CPN1 | .381^*^ | 0.098 | -.386^*^ | -0.11 | -0.087 | -0.026 | 0.102 | -.377^*^ | 0.064 | 0.19 | .419^*^ |
| CPN2 | -0.184 | -0.052 | 0.007 | -0.137 | -.330^*^ | -.431^*^ | -0.093 | -0.132 | 0.044 | 0.01 | 0.153 |
| COLEC12 | 0.035 | 0.015 | 0.045 | 0.035 | -0.065 | -0.095 | 0.054 | -0.089 | .384^*^ | .373^*^ | 0.073 |
| SERPINA1 | .301^*^ | 0.131 | -0.238 | 0.024 | -0.165 | -0.008 | 0.143 | 0.043 | -.301^*^ | -0.082 | -0.003 |
| SERPINA3 | -0.041 | 0.235 | 0.129 | 0.029 | -0.182 | -0.24 | 0.01 | -0.142 | -0.143 | -0.041 | 0.153 |
| SERPINA4 | 0.226 | 0.158 | -.339^*^ | -.289^*^ | 0.267 | 0.279 | 0.059 | -0.264 | 0.081 | 0.168 | 0.239 |
| SERPINA5 | -.466^*^ | 0.048 | .457^*^ | 0.092 | -0.242 | -0.273 | 0.085 | -0.017 | 0.108 | 0 | -0.003 |
| SERPINA6 | -.540^*^ | -0.049 | .395^*^ | -0.022 | -0.143 | -0.112 | 0.194 | -0.063 | 0.069 | -0.183 | 0.025 |
| SERPINA7 | -.337^*^ | 0.137 | .301^*^ | 0.041 | -0.176 | -0.073 | 0.051 | 0.058 | 0.092 | -0.21 | -0.079 |
| SERPINB3 | -0.224 | -0.109 | 0.246 | 0.011 | 0.054 | -0.158 | -.317^*^ | 0.214 | -0.096 | -0.083 | -0.219 |
| SERPINC1 | .607^*^ | .316^*^ | -.404^*^ | -0.034 | 0.29 | 0.289 | 0.031 | -.328^*^ | -0.008 | 0.269 | .358^*^ |
| SERPIND1 | .399^*^ | .342^*^ | -.374^*^ | -0.206 | 0.289 | .362^*^ | 0.043 | -0.193 | 0.046 | 0.037 | 0.175 |
| SERPINF1 | .546^*^ | 0.22 | -.430^*^ | -0.147 | 0.087 | 0.003 | -0.124 | -0.181 | 0.117 | 0.11 | 0.218 |
| SERPINF2 | -0.1 | 0.283 | 0.174 | 0.035 | 0.046 | 0.043 | 0.01 | -.291^*^ | .305^*^ | 0.056 | 0.258 |
| SERPING1 | 0.085 | .295^*^ | -0.049 | -0.119 | -0.056 | -0.023 | -0.031 | -0.239 | -0.035 | -0.066 | 0.243 |

MN, membranous nephropathy; ^*^ *P* < 0.05.

**Supplementary Table 3. Analysis of differences in urinary complement activation in different pathological periods**

| **Urine complements types**  **(Urine complements/all protein)** | **Stage I（=14）** | **Stage II（n=34）** | ***P* Value** |
| --- | --- | --- | --- |
|  |  |  |  |
| C1R | 0 (0, 1.76×10^-4^) | 0 (0, 1.91×10^-5^) | 0.354 |
| C1S | 0 (0, 0) | 0 (0, 0) | 0.359 |
| C2 | 0 (0, 0) | 0 (0, 1.17×10^-5^) | 0.062 |
| C4A | 2.11×10^-4^ (4.85×10^-5^, 4.24×10^-4^) | 6.03×10^-4^ （1.38×10^-4^，2.48×10^-3^) | 0.028 |
| C4B | 0 (0, 1.52×10^-5^) | 1.71×10^-5^ (0, 6.08×10^-5^) | 0.079 |
| C3 | 5.55×10^-4^ (3.10×10^-4^, 2.01×10^-3^) | 1.18×10^-3^ (2.07×10^-4^, 1.14×10^-2^) | 0.563 |
| C6 | 0 (0, 0) | 0 (0, 2.407×10-7) | 0.05 |
| C7 | 0 (0, 1.19×10^-5^) | 1.06×10^-5^（0， 5.58×10^-5^） | 0.087 |
| C8A | 0 (0, 3.01×10^-6^) | 0 (0, 2.62×10^-5^) | 0.396 |
| C8B | 0 (0, 0) | 0 (0, 7.55×10-^-7^) | 0.406 |
| C8G | 0 (0, 0) | 0 (0, 8.47×10^-6^) | 0.114 |
| C9 | 3.52×10^-5^ (0, 2.84×10^-4^) | 1.38×10^-4^ (2.28×10^-5^, 7.10×10^-4^) | 0.106 |
| MASP2 | 1.21×10^-4^ (0, 4.79×10^-4^ ) | 1.19×10^-4^ (2.34×10^-5^, 3.22×10^-4^ ) | 0.785 |
| CLU | 2.00×10^-4^ (1.48×10^-5^, 5.65×10^-4^) | 4.88×1^-4^ (2.29×10^-4^, 1.24×10^-3^) | 0.025 |
| CFD | 0 (0, 0) | 0 (0, 1.29×10-5) | 0.046 |
| CFB | 4×10^-5^ (0, 1.30×10^-4^) | 5.50×10^-5^ (0, 8.12×10^-4^) | 0.384 |
| CFI | 5.47×10^-6^ (0, 4.76×10^-5^) | 1.48×10^-5^ (4.18×10^-6^, 1.24×10^-4^) | 0.105 |
| CFH | 0 (0, 2.50×10^-6^) | 0 (0, 2.81×10^-5^) | 0.136 |
| CFHR1 | 0 (0, 0) | 0 (0, 0) | 0.608 |
| CFHR2 | 0 (0, 0) | 0 (0, 0) | 0.831 |
| CFHR3 | 0 (0, 0) | 0 (0, 0) | 0.097 |
| CD55 | 4.27×10^-5^ (0, 1.15×10^-4^) | 2.80×10^-5^ (0, 1.92×10^-4^) | 1 |
| CD59 | 5.41×10^-4^ (2.21×10^-4^, 8.44×10^-4^) | 4.83×10^-4^ (2.28×10^-4^, 6.77×10^-4^) | 0.691 |
| CD93 | 0 (0, 0) | 0 (0, 0) | 1 |
| VTN | 9.90×10^-5^ (0, 1.68×10^-4^) | 1.29×10^-4^ (2.36×10^-5^, 5.71×10^-4^) | 0.285 |
| COLEC12 | 0 (0, 0) | 0 (0, 0) | 0.541 |
| CPN1 | 0 (0, 0) | 0 (0, 1.36×10^-5^) | 0.01 |
| CPN2 | 1.16×10-5 (0, 4.45×10^-5^) | 2.55×10-5 (0, 4.85×10^-5^) | 0.364 |

**Supplementary Table 4. Analysis of differences in urinary complement activation with and without tubular atrophy/interstitial fibrosis**

| **Urine complements types**  **(Urine complements/all protein)** | **Tubular atrophy/interstitial fibrosis (-) (=21)** | **Tubular atrophy/interstitial fibrosis (+) (=27)** | ***P* Value** |
| --- | --- | --- | --- |
|  |  |  |  |
| C1R | 0 (0, 0) | 0 (0, 0) | 0.725 |
| C1S | 0 (0, 0) | 0 (0, 0) | 0.208 |
| C2 | 0 (0, 1.35×10^-5^) | 0 (0, 0) | 0.224 |
| C4A | 4.21×10-4 (9.81×10-5,2.32×10-3) | 2.74×10^-4^ （1.12×10^-4^，1.22×10^-3^) | 0.611 |
| C4B | 0 (0, 2.09×10^-5^) | 1.29×10^-5^ (0, 5.41×10^-5^) | 0.434 |
| C3 | 6.86×10^-4^ (3.05×10^-4^, 9.08×10^-3^) | 4.09×10^-4^ (1.82×10^-4^, 6.17×10^-3^) | 0.339 |
| C6 | 0 (0, 0) | 0 (0, 0) | 0.642 |
| C7 | 9.13×10^-6^ (0, 5.59×10^-5^) | 5.03×10^-6^ （0， 2.33×10^-5^） | 0.362 |
| C8A | 0 (0, 2.75×10^-5^) | 0 (0, 5.10×10^-6^) | 0.551 |
| C8B | 0 (0, 1.51×10^-6^) | 0 (0, 0) | 0.736 |
| C8G | 0 (0, 2.76×10^-6^) | 0 (0, 0) | 0.65 |
| C9 | 4.71×10^-5^ (2.03×10^-6^, 6.55×10^-4^) | 1.31×10^-4^ (3.50×10^-5^, 3.12×10^-4^) | 0.441 |
| MASP2 | 2.65×10^-5^ (0,1.67×10^-4^) | 1.59×10^-4^ (5.00×10^-5^, 6.68×10^-4^) | 0.04 |
| CLU | 3.60×10^-4^ (1.17×10^-4^, 1.15×10^-3^) | 3.21×10^-4^ (2.01×10^-4^, 6.56×10^-4^) | 0.942 |
| CFD | 0 (0, 9.88×10^-6^) | 0 (0, 4.62×10^-6^) | 0.78 |
| CFB | 6.53×10^-5^ (2.03×10^-6^, 7.13×10^-4^) | 4.98×10^-5^ (0, 1.87×10^-4^) | 0.522 |
| CFI | 0 (0, 2.41×10^-5^) | 1.64×10^-5^ (2.62×10^-6^, 5.19×10^-5^) | 0.551 |
| CFH | 0 (0, 2.91×10^-5^) | 0 (0, 2.22×10^-5^) | 0.548 |
| CFHR1 | 0 (0, 0) | 0 (0, 0) | 0.86 |
| CFHR2 | 0 (0, 0) | 0 (0, 0) | 0.448 |
| CFHR3 | 0 (0, 0) | 0 (0, 0) | 0.814 |
| CD55 | 4.49×10^-6^ (0, 1.09×10^-4^) | 5.90×10^-5^ (0, 2.04×10^-4^) | 0.123 |
| CD59 | 3.68×10^-4^ (1.37×10^-4^, 6.65×10^-4^) | 5.66×10^-4^ (4.26×10^-4^, 9.23×10^-4^) | 0.102 |
| CD93 | 0 (0, 0) | 0 (0, 0) | 1 |
| VTN | 1.20×10^-4^ (0, 3.79×10^-4^) | 1.18×10^-4^ (3.91×10^-5^,1.84×10^-4^) | 0.992 |
| COLEC12 | 0 (0, 0) | 0 (0, 0) | 0.023 |
| CPN1 | 0 (0, 1.03×10^-5^) | 0 (0, 5.24×10^-6^) | 0.804 |
| CPN2 | 1.29×10^-5^ (0, 5.82×10^-5^) | 2.54×10^-5^ (0, 4.50×10^-5^) | 0.817 |

**Supplementary Table 5. Analysis of differences in urinary complement activation with and without renal C3 deposition**

| **Urine complements types**  **(Urine complements/all protein)** | **C3 (-) (=20)** | **C3 (+) (=28)** | ***P* Value** |
| --- | --- | --- | --- |
|  |  |  |  |
| C1R | 0 (0, 1.366×10^-5^) | 0 (0, 0) | 0.044 |
| C1S | 0 (0, 0) | 0 (0, 0) | 0.833 |
| C2 | 0 (0, 0) | 0 (0, 1.02×10^-5^) | 0.165 |
| C4A | 2.40×10^-4^ (3.35×10^-5^,5.16×10^-4^) | 5.01×10^-4^ (1.67×10^-4^，2.12×10^-3^) | 0.083 |
| C4B | 1.53×10^-5^ (0, 3.85×10^-5^) | 5.46×10^-6^ (0, 4.89×10^-5^) | 0.74 |
| C3 | 3.70×10^-4^ (1.67×10^-4^, 4.83×10^-3^) | 9.48×10^-4^ (2.45×10^-4^, 7.88×10^-3^) | 0.291 |
| C6 | 0 (0, 0) | 0 (0, 0) | 0.699 |
| C7 | 5.94×10^-6^ (0, 2.05×10^-5^) | 7.22×10^-6^ (0， 4.65×10^-5^) | 0.746 |
| C8A | 0 (0, 6.05×10^-6^) | 0 (0, 1.04×10^-5^) | 0.75 |
| C8B | 0 (0, 2.83×10^-6^) | 0 (0, 0) | 0.586 |
| C8G | 0 (0, 0) | 0 (0, 4.14×10^-6^) | 0.324 |
| C9 | 4.66×10^-5^ (6.41×10^-6^, 2.68×10^-4^) | 1.48×10^-4^ (3.03×10^-5^, 6.83×10^-4^) | 0.18 |
| MASP2 | 1.40×10^-4^ (5.74×10^-5^,5.77×10^-4^ ) | 5.13×10^-5^ (0, 2.74×10^-4^ ) | 0.097 |
| CLU | 3.07×10^-4^ (1.92×10^-5^, 6.52×10^-4^) | 4.00×10^-4^ (1.75×10^-4^, 1.24×10^-3^) | 0.77 |
| CFD | 0 (0, 4.13×10^-6^) | 0 (0, 3.47×10^-6^) | 0.873 |
| CFB | 3.73×10^-5^ (0, 1.66×10^-4^) | 6.37×10^-5^ (5.02×10^-6^, 7.69×10^-4^) | 0.272 |
| CFI | 1.60×10^-5^ (2.86×10^-6^, 5.12×10^-5^) | 8.00×10^-6^ (6.56×10^-7^, 1.13×10^-4^) | 0.95 |
| CFH | 0 (0, 2.91×10^-5^) | 0 (0, 1.19×10^-5^) | 0.477 |
| CFHR1 | 0 (0, 0) | 0 (0, 0) | 0.906 |
| CFHR2 | 0 (0, 0) | 0 (0, 0) | 0.472 |
| CFHR3 | 0 (0, 0) | 0 (0, 0) | 0.676 |
| CD55 | 6.34×10^-5^ (1.05×10^-5^, 2.70×10^-4^) | 5.88×10^-6^ (0, 1.38×10^-4^) | 0.04 |
| CD59 | 5.89×10^-4^ (4.32×10^-4^, 7.96×10^-4^) | 4.60×10^-4^ (1.83×10^-4^, 7.37×10^-4^) | 0.25 |
| CD93 | 0 (0, 0) | 0 (0, 0) | 1 |
| VTN | 1.30×10^-4^ (1.33×10^-5^, 3.97×10^-4^) | 1.06×10^-4^ (2.10×10^-5^, 2.64×10^-4^) | 0.983 |
| COLEC12 | 0 (0, 0) | 0 (0, 0) | 0.771 |
| CPN1 | 0 (0, 0) | 0 (0, 1.14×10^-5^) | 0.037 |
| CPN2 | 2.36×10^-5^ (0, 4.49×10^-5^) | 2.08×10^-5^ (0, 4.78×10^-5^) | 0.719 |
